# Bacteriophages targeting *Acinetobacter baumannii* in the era of antibiotic failure: a review

**DOI:** 10.3389/fmicb.2026.1778984

**Published:** 2026-03-12

**Authors:** Jamil Allen G. Fortaleza, Kevin Smith P. Cabuhat, Shukho Kim, Ferdinand A. Mortel, Grace D. Bacalzo, Jose Jurel M. Nuevo

**Affiliations:** 1 National University Philippines, Manila, Philippines; 2 Department of Biology, College of Science, De La Salle University, Manila, Philippines; 3 Basic Education Department, La Consolacion University Philippines, Malolos, Philippines; 4 Department of Microbiology, School of Medicine, Kyungpook National University, Daegu, Republic of Korea; 5 College of Medical Technology, Manila Central University, Caloocan, Philippines; 6 College of Allied Medical Sciences, Wesleyan University-Philippines, Cabanatuan City, Philippines; 7 College of Medical Laboratory Science, Our Lady of Fatima University, Valenzuela, Philippines

**Keywords:** biofilm, endolysin, engineered phage, multi-drug resistance, phage-antibiotic combination

## Abstract

*Acinetobacter baumannii* has become a prominent healthcare-associated pathogen due to its exceptional environmental persistence, biofilm-forming capacity, and the global emergence of multidrug-resistant (MDR), extensively drug-resistant (XDR), and pan-drug-resistant lineages. The declining effectiveness of conventional antibiotics has renewed interest in bacteriophage-based strategies as alternative or adjunctive antimicrobial approaches. This review provides a comprehensive synthesis of recent advances in bacteriophages targeting *A. baumannii*, integrating microbiological, structural, immunological, and translational perspectives that shape therapeutic efficacy and resistance dynamics. We highlight the central role of bacterial surface structures, including capsular polysaccharides, outer membrane proteins, and lipooligosaccharides, which act simultaneously as virulence determinants, phage receptors, and modulators of antimicrobial susceptibility. Phage-mediated antibacterial activity is achieved through receptor-specific lysis, biofilm disruption, capsule and envelope remodeling, and indirect resensitization to antibiotics, frequently accompanied by fitness trade-offs in phage-resistant bacterial subpopulations. We further discuss how formulation strategies, phage-derived enzymes, engineered phages, and phage–antibiotic combinations influence treatment outcomes, with particular attention to delivery routes, dosing strategies, and host immune context. Importantly, we distinguish direct phage effects from secondary immunological consequences of bacterial clearance and critically evaluate evidence from *in vitro* studies, animal infection models, and emerging clinical reports. Finally, we examine regulatory and manufacturing challenges that currently limit broader clinical translation. This review positions bacteriophage-based interventions as a promising, evolution-aware complement to antibiotics for managing drug-resistant *A. baumannii*, while underscoring the requirements for their rational and durable clinical implementation.

## Introduction

Over the past decades, *Acinetobacter baumannii* has emerged as a dominant healthcare-associated pathogen, particularly in intensive care units (ICUs) and among immunocompromised patients. Its clinical success is driven by exceptional environmental resilience, including prolonged survival on abiotic surfaces, tolerance to disinfectants, and resistance to physical stressors such as ultraviolet radiation (Peleg et al., 2008; Dijkshoorn et al., 2007). Coupled with its broad ecological distribution in soil, water, air, and on human skin, these traits facilitate both community introduction and sustained nosocomial transmission (Shi et al., 2024). The ability of *A. baumannii* to colonize multiple anatomical niches enables asymptomatic carriage and long-term persistence in hospital environments, promoting contamination of medical devices and environmental reservoirs. These factors collectively underpin recurrent outbreaks of ventilator-associated pneumonia, bloodstream, urinary tract, and wound infections, which are associated with substantial morbidity and mortality (Huang et al., 2018; Dickstein et al., 2019; Decré, 2012; Yun et al., 2018).

The clinical burden of *A. baumannii* infections has been further amplified by the rapid global dissemination of multidrug-resistant (MDR), extensively drug-resistant (XDR), and pan-drug-resistant (PDR) lineages. Reported XDR prevalence ranging from 41 to 93% and the emergence of PDR isolates in up to 3.6% of clinical samples reflect a profound erosion of effective antimicrobial options (Abbasi et al., 2021; Salehi et al., 2018; Sana et al., 2021; Swe-Han et al., 2017; Inchai et al., 2015). These resistance phenotypes translate directly into adverse clinical outcomes, including prolonged ICU stays, increased healthcare costs, and mortality rates exceeding 60% in bacteremia (Pogue et al., 2022). Importantly, the persistence of resistant *A. baumannii* in ICUs is reinforced by modifiable risk factors, such as prior broad-spectrum antibiotic exposure, mechanical ventilation, invasive devices, and extended hospitalization, that create a self-sustaining cycle of selection and transmission (Inchai et al., 2015; Sana et al., 2021; Ren et al., 2019). These dynamics have led the World Health Organization to classify MDR *A. baumannii* as a critical-priority *Enterococcus faecium, Staphylococcus aureus, Klebsiella pneumoniae, A. baumannii, Pseudomonas aeruginosa*, *Enterobacter* spp. pathogen, highlighting the urgent need for alternative therapeutic strategies (WHO, 2024).

The extraordinary adaptability of *A. baumannii* is underpinned by a diverse repertoire of resistance mechanisms that compromise nearly all major antibiotic classes, including β-lactams, aminoglycosides, fluoroquinolones, and last-line agents (Kim et al., 2018; Hameed et al., 2019). In the context of a stagnating antibiotic development pipeline, this resistance landscape has intensified interest in non-traditional antimicrobial approaches, particularly bacteriophage-based interventions. Bacteriophages are highly specific bacterial viruses that exploit host cellular machinery for replication, offering targeted antibacterial activity while largely sparing the commensal microbiota (Reardon, 2014; Fortaleza et al., 2024). However, their therapeutic deployment must account for host immune recognition, pharmacokinetic variability, and the potential release of endotoxins following rapid bacterial lysis, which may exacerbate inflammatory responses in critically ill patients (Hibstu et al., 2022; Emencheta et al., 2024).

From a therapeutic perspective, lytic bacteriophages are generally favored due to their capacity for rapid bacterial killing mediated by coordinated holin-endolysin activity, whereas temperate phages may influence bacterial fitness, virulence, or resistance through lysogenic integration (Gordillo Altamirano and Barr, 2019; Borin et al., 2023; Chen et al., 2014). Accumulating preclinical and clinical evidence supports the feasibility of lytic phage therapy against MDR *A. baumannii*, including compassionate-use cases reported across the United States, Europe, and Eastern Europe (Hon et al., 2022). These reports suggest that phages can complement existing antibiotics and provide therapeutic options in settings where conventional treatments have failed.

Most *A. baumannii* phages characterized to date belong to the order Caudovirales, encompassing the families Myoviridae, Podoviridae, and Siphoviridae, whose structural diversity influences host recognition, adsorption efficiency, and infection dynamics (Jamal et al., 2019; Fortaleza et al., 2025). Effective phage therapy therefore depends on precise receptor recognition, efficient genome delivery, and robust lytic replication, underscoring the importance of rigorous phage isolation, genomic characterization, and safety evaluation (Malik et al., 2017; Stone et al., 2019). Despite growing momentum driven by advances in genomics, bioinformatics, and precision medicine, significant challenges remain, including standardization of manufacturing processes, regulatory harmonization, and integration into routine clinical workflows (Kviatcovsky et al., 2023).

In this review, we critically examine bacteriophage-based interventions as part of a multifaceted strategy to combat antibiotic-resistant *A. baumannii*, focusing on the mechanistic determinants of phage-host interactions, resistance evolution, immune engagement, and the translational and regulatory barriers that currently limit widespread clinical implementation.

## Microbiology of *Acinetobacter baumannii*

*A. baumannii* is a Gram-negative, non-motile, strictly aerobic, non-fermenting coccobacillus belonging to the *A. calcoaceticus-A. baumannii* (Acb) complex, which also includes *A. pittii* and *A. nosocomialis* (Marinova-Bulgaranova et al., 2025; Nurjadi and Boutin, 2022; Sahl et al., 2013). Among these closely related species, *A. baumannii* predominates in hospital environments and accounts for the majority of MDR and XDR infections worldwide, reflecting its exceptional capacity for environmental persistence, dissemination, and acquisition of resistance determinants. The pathogenicity of *A. baumannii* is closely linked to its structural and surface-associated features, which collectively promote host colonization, immune evasion, and persistence. Outer membrane proteins play a central role in these processes: OmpA contributes to biofilm formation, host cell adhesion and invasion, and modulation of host immune responses, whereas Omp33 reduces membrane permeability and enhances virulence (Nie et al., 2020; Abellón-Ruiz et al., 2018). In parallel, the polysaccharide capsule provides protection against phagocytosis and innate immune defenses, representing a key determinant of serum resistance and *in vivo* survival (Luna and Spellberg, 2023; Popova et al., 2021). Type IV pili, particularly PilA, further facilitate adhesion, DNA uptake, and surface-associated motility, enabling colonization of diverse biotic and abiotic niches (Piepenbrink et al., 2016).

Survival under nutrient-limited and hostile host environments is supported by multiple, partially redundant nutrient acquisition systems. Iron uptake is dominated by siderophore-mediated pathways, with the acinetobactin system serving as the primary mechanism. Additional systems, including baumannoferrin, fimsbactin, and TonB-dependent heme and transferrin uptake pathways, provide functional redundancy and enhance fitness under host-imposed iron restriction (Zhang et al., 2025; Sefid et al., 2013). Biofilm formation on medical devices and host tissues further increases tolerance to desiccation, immune clearance, and antimicrobial exposure, contributing to long-term persistence and treatment failure in clinical settings (Tudor et al., 2016; Elkheloui et al., 2020; Jacobs et al., 2010; Zhang et al., 2025). Additional virulence-associated systems, including phospholipase D and the thioredoxin system, support epithelial invasion, serum survival, pilus assembly, and redox homeostasis, reinforcing adaptability during infection (Jacobs et al., 2010; Sefid et al., 2013). Adaptation and antimicrobial resistance in *A. baumannii* are driven by coordinated genetic plasticity and regulatory control. Resistance to β-lactams and carbapenems arises through the combined action of β-lactamase production, efflux pump overexpression, and outer membrane remodeling, with biofilm-associated growth states further enhancing antimicrobial tolerance (Hou and Ying, 2010; Castanheira et al., 2023; Elkheloui et al., 2020). Horizontal gene transfer, natural transformation, and mutational events enable rapid acquisition and dissemination of resistance and virulence traits across clinical lineages (De Silva and Kumar, 2019; Wood et al., 2018).

## Overview of bacteriophage

**Figure 1:**
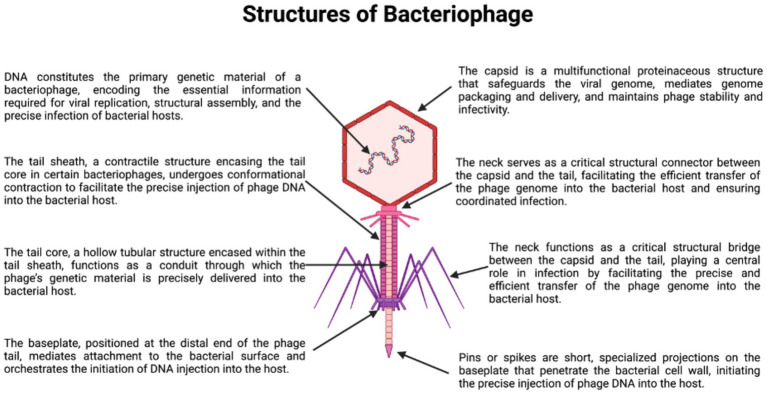
Morphological classes of *A. baumannii* bacteriophages (Fortaleza et al., 2025; Ganeshan and Hosseinidoust, 2019).

Bacteriophages, or phages, are viruses that specifically infect and replicate within bacterial hosts and represent one of the most abundant and diverse biological entities in the biosphere, occurring ubiquitously across aquatic, terrestrial, plant-associated, animal-associated, and wastewater ecosystems (Bisen et al., 2024; Coculescu et al., 2024). Their global distribution reflects a fundamental ecological role in regulating bacterial population dynamics, shaping microbial community structure, and driving bacterial evolution through continuous predator–prey interactions and co-evolutionary pressures (Bisen et al., 2024). Structurally, the majority of bacteriophages infecting clinically relevant bacteria, including *A. baumannii*, belong to tailed phages within the class Caudoviricetes. As summarized in Figure 1, these phages share a conserved architectural framework composed of an icosahedral capsid enclosing the viral genome and a tail apparatus responsible for host recognition and genome delivery. Despite this shared organization, substantial variation exists in tail length, flexibility, and contractility, features that critically influence adsorption kinetics, receptor engagement, and infection efficiency. Myovirus-like phages possess long, contractile tails that enable forceful genome injection, whereas siphovirus- and podovirus-like phages rely on non-contractile tails typically associated with more selective receptor recognition and narrower host ranges (Arivarasan and Consul, 2023; Sanz-Gaitero et al., 2021).

**Figure 2:**
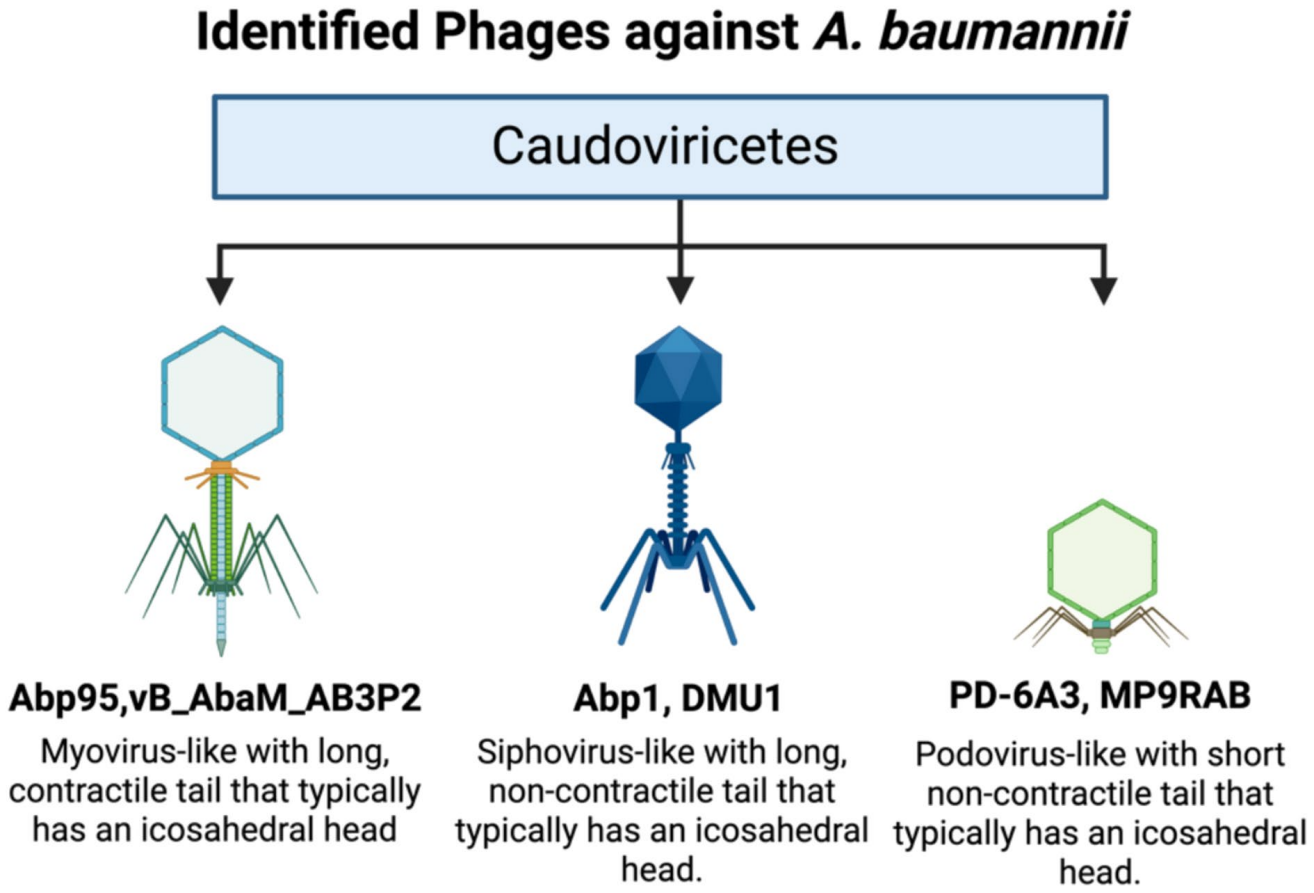
Taxonomic and morphological diversity of bacteriophages infecting *A. baumannii* (Veesler and Cambillau, 2011; Elbreki et al., 2014).

Building upon this conserved structural blueprint, bacteriophages infecting *A. baumannii* exhibit substantial functional and genomic diversity, with representative examples summarized in Figure 2 illustrating how morphological variation translates into therapeutic potential. Recent investigations have further expanded this repertoire through the isolation and characterization of novel lytic bacteriophages specifically active against carbapenem-resistant A. baumannii. For example, newly identified phages with strong lytic activity and favorable biological properties have been reported to effectively target clinical carbapenem-resistant isolates, highlighting the ongoing discovery of therapeutically relevant phages for combating multidrug-resistant *A. baumannii* (Choi et al., 2024). Phage Abp95, a myovirus-like phage characterized by a contractile tail, displays a relatively narrow host range; however, its short latency period, high burst size, rapid adsorption kinetics, and the presence of an encoded capsular depolymerase enhance lytic efficiency and support its candidacy for targeting carbapenem-resistant *A. baumannii* (Huang et al., 2023). In contrast, phage Abp1 possesses a typical icosahedral head and a prominent, slender, non-contractile tail consistent with siphovirus-like morphology. Its genome, approximately 40–50 kb in size, encodes multiple proteins associated with host adsorption and biofilm penetration, enabling high specificity toward the *A. baumannii* AB1 strain (Wang et al., 2025a, b). Additional members of the Caudoviricetes further underscore the functional breadth of *A. baumannii*-infecting phages. Friunavirus phage PD-6A3 maintains strong lytic activity across a wide temperature range (4–50 °C) and pH spectrum (5–10), achieving more than 90% host adsorption within 5 min, properties indicative of rapid infection dynamics and notable environmental resilience (Wu et al., 2019). Similarly, phage MRABP9 demonstrates a short latency period, high burst yield, pronounced antibiofilm activity, and effective suppression of bacterial regrowth while retaining excellent physicochemical stability (Zhang et al., 2024). These characteristics highlight the adaptive capacity of *A. baumannii* phages and reinforce their suitability for therapeutic development, particularly in clinical contexts where biofilm disruption, environmental stability, and rapid bacterial clearance are critical. Collectively, the structural versatility and functional adaptability of these phages provide a strong foundation for the advancement of precision phage-based strategies against multidrug-resistant pathogens.

## Cellular structures of *Acinetobacter baumannii* relevant to phage therapy

The success of bacteriophage therapy against *A. baumannii* is strongly influenced by the structural composition and variability of bacterial surface components that mediate phage adsorption, infection, and resistance. Key cellular structures, including capsular polysaccharides, outer membrane proteins, and lipooligosaccharides, play dual roles in virulence and antimicrobial resistance while simultaneously acting as critical determinants of phage specificity and therapeutic efficacy.

### Capsular polysaccharides

Capsular polysaccharides (CPS) are central virulence determinants of *A. baumannii*, mediating immune evasion, biofilm formation, serum resistance, and serving as primary receptors for bacteriophage adsorption (Sianturi et al., 2022; Chen et al., 2023; Shan et al., 2022). Extensive CPS structural heterogeneity arises from variation in monosaccharide composition, glycosidic linkages, and polymer architecture, which are encoded by highly diverse K locus (KL) gene clusters (Zou et al., 2024; Arbatsky et al., 2024; Arbatsky et al., 2018; Arbatsky et al., 2015). Even closely related strains can exhibit distinct capsular conformations; for example, strains MAR 15-4076 (KL129) and LUH5540 (KL84) share identical pentasaccharide repeat units yet differ in overall capsule topology due to variation in the Wzy polymerase, resulting in functionally distinct CPS architectures (Arbatsky et al., 2024). Such fine-scale structural variation has profound consequences for immune recognition, complement resistance, and susceptibility to phage infection.

CPS biosynthesis is orchestrated by KL-encoded enzymatic machinery, including glycosyltransferases, flippases, and polymerases. For instance, KL125 encodes three glycosyltransferases that are indispensable for assembly of the K125 capsule (Arbatsky et al., 2018). At a global regulatory level, the BfmRS two-component system acts as a master regulator of envelope biogenesis, modulating expression of more than 1,000 genes involved in capsule production, peptidoglycan synthesis, outer membrane remodeling, and surface appendages (Raustad et al., 2025; Russo et al., 2016; Geisinger et al., 2018; Palethorpe et al., 2022). Through this broad regulatory scope, BfmRS links CPS expression to biofilm formation, siderophore-mediated iron acquisition, type IV pili assembly, and stress adaptation, thereby coordinating persistence, antibiotic tolerance, and survival under hostile clinical conditions (Draughn et al., 2018; Lokhande et al., 2023; Krasauskas et al., 2019).

From a therapeutic perspective, CPS represents the dominant adsorption receptor for many *A. baumannii*–specific bacteriophages. Numerous CPS-targeting phages encode tailspike depolymerases (TSDs) or related polysaccharide-degrading enzymes that selectively recognize and dismantle specific CPS types, thereby enabling phage attachment and genome delivery. For example, the myovirus TaPaz infects K47 strains through two TSDs, including a glycosidase that cleaves the K47 capsule (Shchurova et al., 2021). Similarly, friunaviruses such as APK09, APK14, APK16, APK37.1, APK86, APK127v, and APK128 exhibit strict CPS-type specificity toward K9, K14, K16, K37/K3-v1, K86, K127, and K128, respectively (Timoshina et al., 2023), while phage Aristophanes targets K26 via a tailspike-associated deacetylase (Timoshina et al., 2021). CPS degradation is mediated by either hydrolytic glycosidases or polysaccharide lyases, as exemplified by phage AP22 (Knirel et al., 2020).

These highly specific CPS-phage interactions highlight both the therapeutic promise and inherent limitations of phage-based strategies against drug-resistant *A. baumannii*. While CPS-targeting phages and depolymerases offer precise, potent antibacterial activity (Chen et al., 2022), their narrow host range and the rapid emergence of CPS-modified, phage-resistant variants necessitate comprehensive phage libraries and rationally designed phage cocktails. Addressing the extraordinary CPS diversity of *A. baumannii* will therefore be critical for achieving durable and broadly effective phage therapy (Timoshina et al., 2023; Islam et al., 2024).

### Outer membrane proteins

Outer membrane proteins (OMPs) are central mediators of virulence, environmental fitness, and therapeutic responsiveness in *A. baumannii*, coordinating host adhesion, invasion, biofilm formation, immune evasion, and antimicrobial susceptibility (Abellón-Ruiz et al., 2018; Nie et al., 2020). Among these, AbOmpA is the most extensively characterized OMP and functions as a β-barrel porin with an N-terminal transmembrane domain and a C-terminal periplasmic region that stabilizes the outer membrane, supports envelope integrity, and facilitates host–pathogen interactions (Zhao et al., 2024). Beyond OmpA, additional OMPs such as CarO, BauA, and OprD-like porins play critical roles in carbapenem permeability, siderophore-mediated iron uptake, and multidrug resistance, collectively shaping both pathogenic potential and treatment outcomes (Mussi et al., 2007; Ramezanalizadeh et al., 2021). Notably, isolate-specific variation in OMP composition and expression contributes to phenotypic heterogeneity, influencing virulence, antibiotic susceptibility, and vulnerability to bacteriophage infection.

OMP expression is controlled by multilayered regulatory networks that integrate environmental sensing with envelope remodeling. Two-component systems such as BfmRS and PmrAB coordinate transcriptional responses that affect capsule synthesis, membrane permeability, and stress adaptation, while global regulators and RNA-binding proteins, including Hfq, fine-tune OMP abundance at the post-transcriptional level (Kim et al., 2019; Palethorpe et al., 2022; Ko et al., 2023; Park et al., 2021; Oh et al., 2020; Kuo et al., 2017). Additional modulation occurs through metal-responsive regulators such as Zur, which controls lipoproteins including ZrlA; loss of ZrlA enhances outer membrane vesicle (OMV) production and cytotoxicity, further illustrating how OMP regulation intersects with virulence and immune interaction (Kim et al., 2021).

From a therapeutic perspective, OMPs serve as key bacteriophage receptors and thus critically shape phage host range and infection dynamics. Recent work demonstrates that phages StAb1, StAb2, and StAb3 exploit distinct entry pathways, including capsule-dependent adsorption, CarO-mediated binding, and interaction with conserved phage glycan receptors (PGRs), enabling differential access to *A. baumannii* populations with variable surface architectures (McCalla et al., 2025). Importantly, phage resistance frequently arises through downregulation or structural modification of OMPs, most notably OmpA, thereby reducing phage adsorption. Such resistance, however, is often accompanied by pleiotropic fitness costs, including altered biofilm formation, reduced motility or virulence, and increased susceptibility to antibiotics, highlighting exploitable evolutionary trade-offs (Nayak et al., 2025).

Despite increasing recognition of their importance, many *A. baumannii* OMPs remain poorly characterized, particularly with respect to structural diversity, regulatory plasticity, and phage interaction specificity. Addressing these knowledge gaps will be essential for rational phage selection, design of phage-antibiotic combination therapies, and development of host-targeted strategies that exploit envelope vulnerabilities. A deeper mechanistic understanding of OMP biology therefore represents a critical frontier for advancing durable, evolution-informed interventions against multidrug-resistant *A. baumannii* (Gordillo Altamirano and Barr, 2019; Palethorpe et al., 2022; Alquethamy et al., 2022).

### Lipooligosaccharides

Lipooligosaccharides (LOS) are integral surface components of *A. baumannii* that play critical roles in virulence, immune modulation, and susceptibility to bacteriophage infection. Unlike classical lipopolysaccharides (LPS), LOS lacks a repeating O-antigen and consists of a lipid A moiety linked to a core oligosaccharide. Lipid A, a diphosphorylated disaccharide substituted with multiple fatty acyl chains, is the principal determinant of endotoxic activity and host inflammatory signaling (Fregolino et al., 2010; Li et al., 2025). Structural variability within the oligosaccharide backbone, including charge-altering and zwitterionic modifications, further enhances immune evasion, membrane stability, and pathogenic fitness (Li et al., 2025). The genes governing LOS biosynthesis are localized between *ilvE* and *aspS*, and extensive diversity within the outer core (OC) locus generates distinct LOS chemotypes that influence virulence, immune recognition, and antimicrobial susceptibility (Kenyon et al., 2014).

Functionally, LOS represents a dynamic interface between phage predation and bacterial defense. LOS expression is closely coordinated with CPS synthesis encoded at the K locus, such that reversible genetic alterations can simultaneously remodel capsule and LOS architecture. This coupling has important therapeutic consequences: phages preferentially target encapsulated phenotypes, whereas non-encapsulated, phage-resistant variants exhibit increased susceptibility to complement-mediated killing, resulting in an emergent synergistic antibacterial effect driven by host immunity (Timoshina et al., 2023; Popova et al., 2021). Although direct evidence remains limited, lipid A and core oligosaccharide diversification are likely to influence phage adsorption efficiency by altering membrane charge, receptor accessibility, and envelope rigidity, thereby shaping phage-host co-evolution during therapy.

Beyond this indirect role, LOS can also function as a direct phage receptor. Several *A. baumannii* phages encode depolymerases or tail fiber-associated enzymes capable of degrading LOS to facilitate adsorption and genome delivery. For example, the depolymerase Dpo71 disrupts LOS structure, sensitizing bacteria to colistin and impairing biofilm formation (Chen et al., 2022), while phage ISTD produces LOS-targeting depolymerases that effectively reduce both planktonic and biofilm-associated populations (Vukotic et al., 2020). In addition to enhancing phage infectivity, LOS degradation products can modulate innate immune responses, including macrophage activation, highlighting a potential immunological dimension to LOS-directed phage therapy (Huang et al., 2026).

## Phage formulations used in treatments

The clinical effectiveness of phage therapy is strongly influenced by formulation strategy, which determines therapeutic breadth, resistance suppression, stability, safety, and regulatory feasibility. To date, whole-phage preparations remain the most mature and widely applied modality. Monophage therapy, which employs a single, highly specific phage, offers conceptual simplicity, precise targeting, and relatively straightforward manufacturing and quality control. However, its clinical utility is constrained by narrow host range and vulnerability to rapid resistance emergence in heterogeneous or rapidly evolving bacterial populations (Eyer et al., 2017; Teklemariam et al., 2024). To overcome these limitations, phage cocktails combining multiple phages with distinct receptor specificities are frequently employed to broaden antibacterial coverage and suppress resistance development (Chan and Abedon, 2012; Teklemariam et al., 2024). Both experimental and clinical data indicate that cocktails improve robustness against strain diversity and delay resistance emergence. Concerns regarding dysbiosis largely remain theoretical, as phages exhibit far narrower host ranges than antibiotics, although broader cocktails may unintentionally affect closely related commensal bacteria. Similarly, risks related to horizontal gene transfer are primarily associated with temperate phages rather than strictly lytic preparations, emphasizing that these risks are context-dependent and can be mitigated through rational phage selection, genomic screening, and regulatory oversight rather than representing inherent limitations of cocktail formulations (Teklemariam et al., 2024).

Beyond naturally occurring phages, genetically modified and non-replicating phages have emerged as advanced formulations designed to enhance safety, control, and therapeutic precision. Engineered phages may be modified to remove lysogeny-associated genes, eliminate replication capacity, or alter receptor-binding domains, thereby reducing risks of uncontrolled *in vivo* amplification and horizontal gene transfer (Matsuzaki et al., 2005). Non-replicating phages function as targeted antibacterial delivery vehicles rather than self-amplifying agents, offering predictable pharmacokinetics and improved regulatory tractability. Compared with natural phages, engineered variants provide enhanced targeting specificity, reduced evolutionary unpredictability, and compatibility with adjunctive payloads such as enzymes or antimicrobial molecules. However, most genetically modified phages remain at the preclinical or early experimental stage, with limited clinical documentation to date, rendering their clinical readiness lower than that of lytic natural phages. Nonetheless, they represent a strategically important platform for addressing safety and regulatory barriers that complicate broader clinical adoption of conventional phage therapy.

In addition to intact phages, phage-derived components, including lysins and other phage-encoded proteins, represent an alternative formulation approach. These agents lack replicative capacity and can be dosed similarly to conventional biologics, offering advantages in manufacturing consistency and regulatory classification. While lysins are most effective against Gram-positive bacteria, engineered variants and combination strategies have expanded their relevance to Gram-negative pathogens (Matsuzaki et al., 2005; Maciejewska et al., 2018). Such components are best viewed as adjunctive therapies that complement antibiotics or whole-phage approaches rather than replacing them, particularly in settings where precise dosing and predictable pharmacodynamics are prioritized.

Formulation effectiveness is also tightly linked to delivery platform and dosage form. Liquid preparations remain the most widely used due to ease of production and compatibility with topical, oral, and intravenous administration, particularly in clinical trials (Chang et al., 2022; Uyttebroek et al., 2023). However, liquid formulations are prone to titer loss during storage and transport. Semi-solid formulations, including gels, creams, and ointments, are especially advantageous for topical treatment of wounds and burns, enabling localized delivery while protecting phages from environmental stressors (Chang et al., 2020). Encapsulated formulations using liposomes or polymeric nanoparticles further enhance stability and targeting by protecting phages from gastric acidity and enabling controlled release at infection sites (Chang et al., 2020; Rosner and Clark, 2021). Phage-immobilized dressings provide sustained local delivery in chronic wounds and diabetic ulcers, maintaining high phage concentrations while minimizing systemic exposure (Chang et al., 2020). Personalized magistral preparations, tailored to individual patients and infecting strains, are clinically documented in compassionate-use settings and exemplify the adaptability of phage therapy in practice (Krylov et al., 2019; Verbeken and Pirnay, 2022).

**Figure 3:**
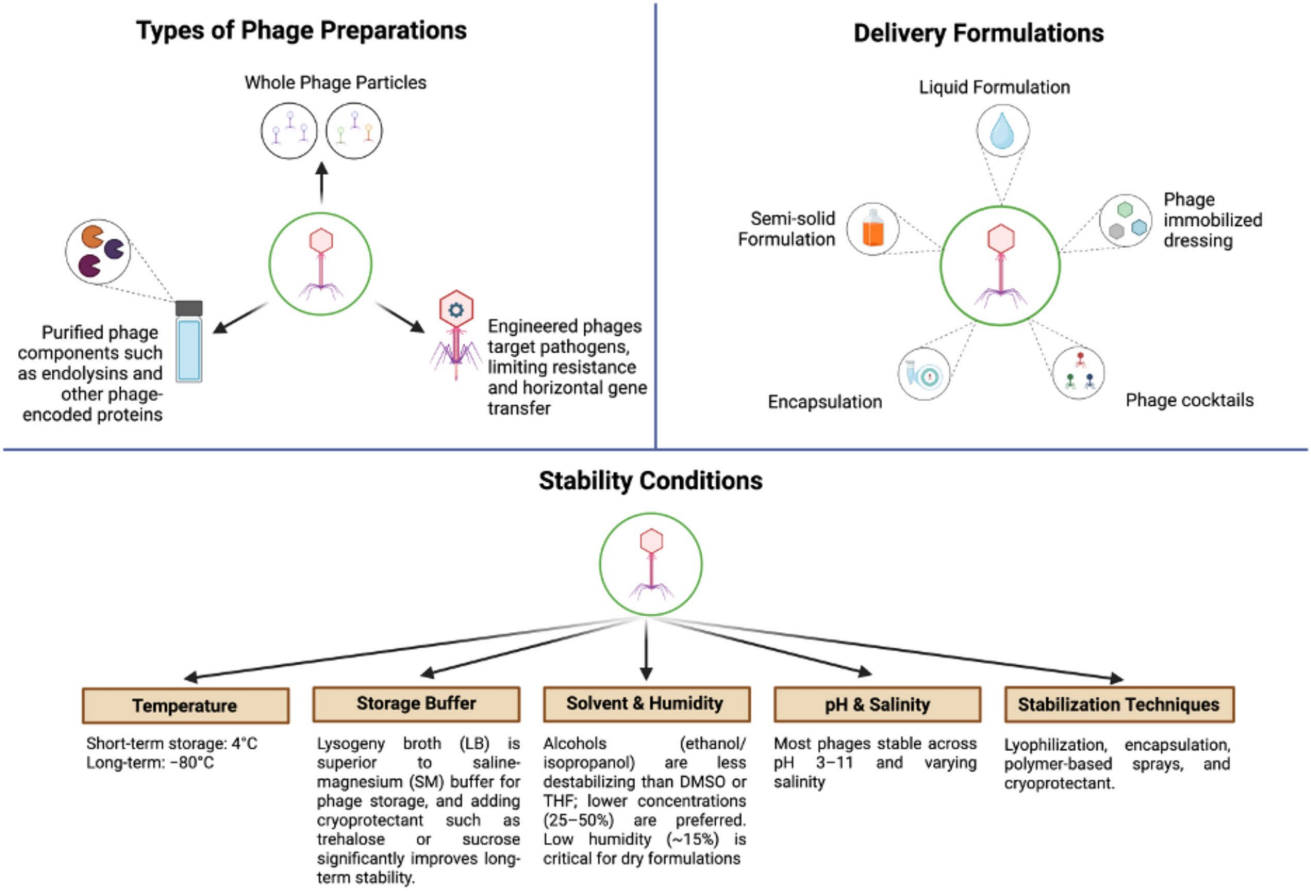
Schematic overview of bacteriophage-based therapeutic approaches (Cha et al., 2018; Fortaleza et al., 2025).

Therapeutic success is further dependent on stability and storage conditions. Phages generally remain stable at 4 °C for short-term storage and −80 °C for long-term preservation, whereas elevated temperatures can rapidly reduce viability through protein denaturation and nucleic acid degradation (Jepson and March, 2004; Chang et al., 2022). Buffer composition influences stability, with lysogeny broth often outperforming saline–magnesium buffer, and cryoprotectants such as trehalose or sucrose significantly improving preservation during freeze-drying (Jepson and March, 2004; Xiao et al., 2022). Environmental factors also modulate viability: alcohols such as ethanol and isopropanol are less destabilizing than solvents like dimethyl sulfoxide or tetrahydrofuran at low concentrations (Cao et al., 2023), while high relative humidity negatively affects dry formulations. Most phages tolerate a broad pH range (3–11) and variable salinity, although extreme conditions lead to inactivation (Li et al., 2025; Wdowiak et al., 2022). Figure 3 presents a schematic overview of bacteriophages, highlighting the major types of phage preparations (whole phage particles, purified components, and engineered phages), delivery formulations (liquid, semi-solid, encapsulated systems, phage-immobilized dressings, and phage cocktails), and the key determinants influencing stability. Stability parameters include temperature, storage buffer composition, solvent exposure and humidity, pH and salinity, and stabilization techniques such as lyophilization, encapsulation, polymer-based formulations, and cryoprotectant use.

## Application of phage therapy in *Acinetobacter baumannii* infections

In pulmonary infections, among the most severe manifestations of *A. baumannii* disease, multiple lytic phages have demonstrated strong antibacterial activity both *in vitro* and *in vivo*, using models such as murine and *G. mellonella* larvae. Treatment with phages such as vB_AbaM_Acibel004 and vB_AbaM_ABPW7 resulted in marked reductions in pulmonary bacterial burden, disruption of biofilms, attenuation of lung inflammation, and improved tissue integrity (Wintachai et al., 2022a). Available evidence suggests that reductions in pro-inflammatory markers and lung injury are largely secondary to rapid bacterial clearance rather than direct phage-mediated immunomodulation. Advances in formulation science, including inhalable phage powders, further enhance the feasibility of localized, non-invasive delivery while mitigating pharmacokinetic limitations associated with systemic antibiotic therapy (Wintachai and Voravuthikunchai, 2022). Beyond respiratory disease, phage therapy has shown consistent efficacy in wound and soft tissue infection models, where biofilm formation frequently drives chronic infection and treatment failure. Diverse phages reduced bacterial loads, accelerated wound closure, and improved healing outcomes without detectable systemic toxicity (Sherif et al., 2025; Wintachai and Voravuthikunchai, 2022). Several candidates, including vWUPSU and vABWU2101, demonstrated potent antibiofilm activity by inhibiting biofilm formation and disrupting mature biofilms (Wintachai et al., 2019; Mukhopadhyay et al., 2023). Rather than acting in isolation, phages often exhibited synergistic interactions with antibiotics; combinations involving phage YC#06 and β-lactams or chloramphenicol achieved greater biofilm reduction than either modality alone (Luo et al., 2024; Hussain et al., 2023). Sustained local delivery platforms, including phage-loaded hydrogels, further enhanced therapeutic efficacy and emphasize the translational importance of formulation-driven optimization for complex soft tissue infections (Ghaznavi-Rad et al., 2022; Kusradze et al., 2016).

Systemic infections caused by XDR *A. baumannii*, including bacteremia and severe pneumonia, remain the most critical therapeutic challenge due to high mortality and limited treatment options. Experimental studies demonstrate that early or concurrent phage administration confers near-complete protection in murine bacteremia models, whereas delayed treatment yields reduced but still meaningful survival benefits, underscoring the importance of treatment timing (Caflisch et al., 2019). Additionally, lytic phages, including vB_AbaM_3054, vB_AbaM_3090, and vB_AbaS_qsb1, have shown protective efficacy in *G. mellonella* and murine models, reinforcing the therapeutic breadth of phage-based interventions beyond individual candidates (Leshkasheli et al., 2019; Rastegar et al., 2024a,b). Notably, phage-resistant mutants emerging during treatment frequently exhibit reduced fitness, impaired virulence, or increased antibiotic susceptibility, revealing evolution-associated trade-offs that may be strategically exploited through rational combination therapy. Across infection models, dosing strategy and treatment timing consistently emerge as dominant determinants of therapeutic success. Prophylactic or simultaneous administration of phage cocktails (ɸAb4, ɸAb7, ɸAb14) completely prevented mortality in immunocompromised mice, while delayed or reduced-dose regimens still conferred partial protection (Patel et al., 2021). Collectively, these findings support the view that phage therapy is best understood not as a single-agent intervention but as a platform technology whose efficacy depends on rational phage selection, formulation, timing, and integration with antibiotics.

Altogether, the compiled evidence positions phage therapy as a potent, evolution-aware, and clinically adaptable strategy capable of delivering robust antibacterial and antibiofilm activity across pulmonary, wound, and systemic *A. baumannii* infections. While the growing number of *in vivo* studies is encouraging, variability in experimental design, infection models, and dosing regimens highlights the need for standardized protocols to strengthen translational confidence. Nevertheless, when deployed within optimized formulations and guided by mechanistic insight into phage-host interactions, bacteriophage therapy represents one of the most promising strategies currently available to counter the escalating global threat posed by MDR and XDR *A. baumannii* (de la Noue et al., 2025; Pasharawipas et al., 2025). To facilitate cross-study comparison and provide a consolidated overview of therapeutic candidates, key bacteriophages, their bacterial targets, mechanistic attributes, and levels of supporting evidence are summarized in Table 1.

**Table 1:** Representative of bacteriophages targeting multidrug-resistant *A. baumannii.*

Bacteriophage	Bacterial target/strain	Primary antibacterial mechanisms	Experimental model	Reference(s)
vB_AbaM_Acibel004	MDR and clinical isolates of *A. baumannii*	Strictly lytic replication; biofilm disruption; enhances antibiotic susceptibility; reduces infection-associated inflammation secondary to bacterial clearance	*in vivo*: Murine lung infection model	Wienhold et al. (2021)
vB_AbaM_ABPW7	MDR *A. baumannii*	Prevents biofilm formation and eradicates established biofilms	*in vitro*: Crystal violet assay	Wintachai et al. (2022)
vWUPSU	MDR *A. baumannii*	Inhibits biofilm formation; disrupts mature biofilms; synergistic activity with sacha inchi oil	*in vitro*: Crystal violet assay	Wintachai and Voravuthikunchai (2022)
vABWU2101	MDR *A. baumannii*	Biofilm inhibition and eradication; synergizes with antibiotics against planktonic and biofilm-associated bacteria	*in vitro*: Crystal violet assay, fluorescence microscopy	Mukhopadhyay et al. (2023)
YC#06	Multiple clinically relevant MDR *A. baumannii* strains	Lytic activity; biofilm inhibition; phage–antibiotic synergy (chloramphenicol, imipenem, cefotaxime)	*in vitro*: Plaque assay, one-step growth, PAS, time-kill, biofilm assays; *in vivo*: Zebrafish infection model	Luo et al. (2022) and Hussain et al. (2023)
vB_AbaM_3054	XDR *A. baumannii*	High therapeutic efficacy; reduces mortality and infection severity	*in vivo*: *G. mellonella* and murine models	Leshkasheli et al. (2019)
vB_AbaM_3090	XDR *A. baumannii*	High therapeutic efficacy; reduces mortality and infection severity	*in vivo*: *G. mellonella* and murine models	Leshkasheli et al. (2019)
vB_AbaS_qsb1	*A. baumannii* ioag01	Rapid bacterial lysis; anti-biofilm and depolymerase activity; reduces infection-induced inflammation	*in vivo*: Murine pneumonia model	Wang et al. (2025a, b)
ɸAb4, ɸAb7, ɸAb14 (cocktail)	*A. baumannii*	Time- and dose-dependent efficacy; prophylactic administration prevents mortality; delayed treatment reduces death	*in vivo*: Murine (Swiss albino mice)	Patel et al. (2021)
Ab105-2phiΔCI404ad	Clinical strain of *A. baumannii*	Strong antibiofilm activity	*in vitro*: Crystal violet assay	Blasco et al. (2022)

MDR, multiple drug-resistant; XDR, extensively drug-resistant.

## Phage-derived depolymerases, endolysins, and engineered phages targeting *Acinetobacter baumannii*

Phage-derived depolymerases are specialized enzymes that degrade extracellular polysaccharide structures, most notably capsular polysaccharides and, in some cases, components of the biofilm matrix. By dismantling these protective barriers, depolymerases weaken biofilm architecture, enhance phage adsorption, and increase bacterial susceptibility to host immune mechanisms and adjunctive antimicrobial therapies (Nazir et al., 2023). Their activity is typically capsule-type specific, conferring high precision but also limiting standalone host range. Several depolymerases have demonstrated strong antibiofilm and therapeutic potential. Dp49, derived from phage IME285, exhibits activity against 25 of 49 tested *A. baumannii* strains and significantly enhances serum-mediated bacterial killing *in vitro* while improving survival in murine infection models (Wang et al., 2020). Similarly, DepAPK09 displays high physicochemical stability and marked efficacy in murine sepsis and burn wound models, where it reduces bacterial burden and disrupts both developing and mature biofilms (Borzilov et al., 2025). The depolymerase 31TSP further illustrates the robustness of enzyme-based approaches, retaining activity across wide pH and temperature ranges and enhancing biofilm inhibition when combined with ampicillin (Zhang et al., 2025). In these contexts, enhanced antibacterial effects are best interpreted as permissive or enabling, whereby capsule degradation restores antibiotic penetration or immune access rather than producing direct bactericidal synergy.

Phage-derived endolysins also represent a promising class of non-replicative antibacterials, although their activity against *A. baumannii* must be interpreted considering the Gram-negative outer membrane barrier, which restricts access to the peptidoglycan layer. Effective endolysin activity therefore typically requires membrane permeabilization through adjunctive agents, formulation strategies, or specific biochemical conditions (Islam et al., 2023). The N-acetylmuramidase ABgp46 reduces MDR *A. baumannii* populations by approximately 2 log units within 2 h, with expanded antibacterial activity observed in the presence of organic acids such as citric and malic acid (Oliveira et al., 2016). In this case, organic acids likely function as outer membrane destabilizers, facilitating enzyme access rather than generating true synergistic killing. Similarly, LysAB2 demonstrates enhanced antibacterial and antibiofilm activity when combined with colistin, an antibiotic known to disrupt the outer membrane, with efficacy validated in a *G. mellonella* infection model (Zhang et al., 2024). In addition, the endolysin LysSS has been reported to exhibit direct and potent lytic activity against clinical isolates of multidrug-resistant *A. baumannii* as well as *Pseudomonas aeruginosa*, demonstrating broad-spectrum antibacterial potential and supporting its utility as a therapeutic enzyme candidate (Kim et al., 2020). LysAB1245 exhibits broad-spectrum antibacterial activity and significant biofilm reduction against *A. baumannii*, as confirmed by scanning electron microscopy and confocal laser scanning microscopy (Soontarach et al., 2024). Collectively, these findings indicate that endolysins function most effectively as adjunctive agents, whose activity depends on permissive membrane-disrupting conditions rather than intrinsic potency alone.

Engineered bacteriophages represent a mechanistically distinct and increasingly sophisticated extension of phage-based therapeutics. Unlike depolymerases and endolysins, engineered phages retain replicative capacity while incorporating novel functional modules designed to enhance antibiofilm efficacy, expand host range, or suppress resistance (Traore et al., 2025). A notable example is the phage-photosensitizer conjugate ABP-Ce6, in which chlorin e6 functionalization enables localized reactive oxygen species generation upon phage binding. This targeted delivery strategy effectively disrupts biofilms and kills carbapenem-resistant *A. baumannii* (CRAB), including phage-resistant strains, by coupling phage specificity with photodynamic antimicrobial action (Su et al., 2025). Importantly, this approach bypasses classical receptor-based resistance by introducing a non-receptor-dependent killing mechanism, although long-term stability and host tissue safety remain important translational considerations.

Genetic engineering strategies such as tail fiber recombination further expand phage host range by enabling recognition of alternative surface receptors across genetically diverse *A. baumannii* populations (Wang et al., 2025a, b). While host-range expansion improves immediate therapeutic coverage, its evolutionary durability remains uncertain, as broader receptor targeting may impose stronger selective pressure for receptor modification or loss. Long-term stability, resistance counter-selection, and fitness costs associated with such engineered phages therefore require systematic evaluation. Phage cocktails represent an additional strategy to mitigate resistance and broaden efficacy. A cocktail of four temperate phages (SA1, Eve, Ftm, and Gln) effectively inhibited and degraded biofilms formed by XDR *A. baumannii*, with enhanced eradication observed when combined with antibiotics (Rastegar et al., 2024a; Grygorcewicz et al., 2021). Additional studies in human urine models demonstrate that phage cocktails reduce biofilm biomass and improve antibiotic performance under both sequential and simultaneous treatment regimens (Grygorcewicz et al., 2023; Erol et al., 2023). However, the use of temperate phages introduces regulatory and biosafety considerations, including risks of lysogeny and horizontal gene transfer, underscoring the need for careful phage selection and rational engineering.

## Phage resistance and mitigation strategies in *Acinetobacter baumannii*

One of the most extensively characterized mechanisms of phage resistance in *A. baumannii* involves capsule modulation, as capsular polysaccharides frequently serve as primary receptors for bacteriophage adsorption. Genetic alterations affecting capsule biosynthesis, including mutations in the global regulatory system BfmRS and in capsule-associated glycosyltransferases, can substantially reduce phage binding and confer resistance (Bai et al., 2023). These capsule-centered resistance strategies are particularly relevant given the high structural diversity and phase variability of *A. baumannii* capsules, which enable rapid phenotypic adaptation under phage selective pressure (Karamlou et al., 2025). Importantly, resistance is not exclusively capsule-dependent, and reliance on capsule-targeting phages alone may therefore be insufficient for durable therapeutic control. Capsule-independent resistance pathways have emerged as a critical consideration, as they allow *A. baumannii* to evade phage infection without altering capsular architecture (Peng et al., 2024). This is exemplified by rapid resistance to the phages LemonAid and Tonic, mediated by an 8 bp insertion in a gene encoding a previously uncharacterized surface protein proposed to function as a novel phage receptor, occurring in the absence of detectable capsule modification (Manley et al., 2024). This observation highlights the capacity of *A. baumannii* to exploit alternative surface structures for phage evasion and underscores why resistance mechanisms cannot be fully explained by capsule dynamics alone.

Additional resistance strategies operate at multiple biological levels. Receptor-level modifications, including alterations that impair phage tail spike binding, can directly prevent adsorption (Domingues et al., 2021). Biofilm formation further limits phage penetration by creating spatial refuges that shield subpopulations from infection, enabling persistence even in the presence of active phages (Dong et al., 2013). Outer membrane remodeling and altered efflux pump activity have also been implicated in resistance, potentially by modifying surface accessibility or physicochemical properties that influence phage attachment and entry; however, these mechanisms remain less well defined in *A. baumannii* and likely contribute to a strain- and context-dependent manner (Hou and Ying, 2010). CRISPR-Cas–mediated adaptive immunity, which enables sequence-specific degradation of invading phage genomes, has been identified in *A. baumannii* but appears to play a more limited and variable role compared with receptor- and surface-based resistance mechanisms (Scarrone et al., 2025). While CRISPR-Cas systems can confer strong resistance under certain conditions, their uneven distribution across clinical isolates and frequent absence in highly resistant hospital lineages suggest that CRISPR-Cas is not a dominant or universal barrier to phage therapy in *A. baumannii*. Its contribution should therefore be viewed as complementary rather than central in the resistance landscape (Joseph et al., 2025).

**Figure 4:**
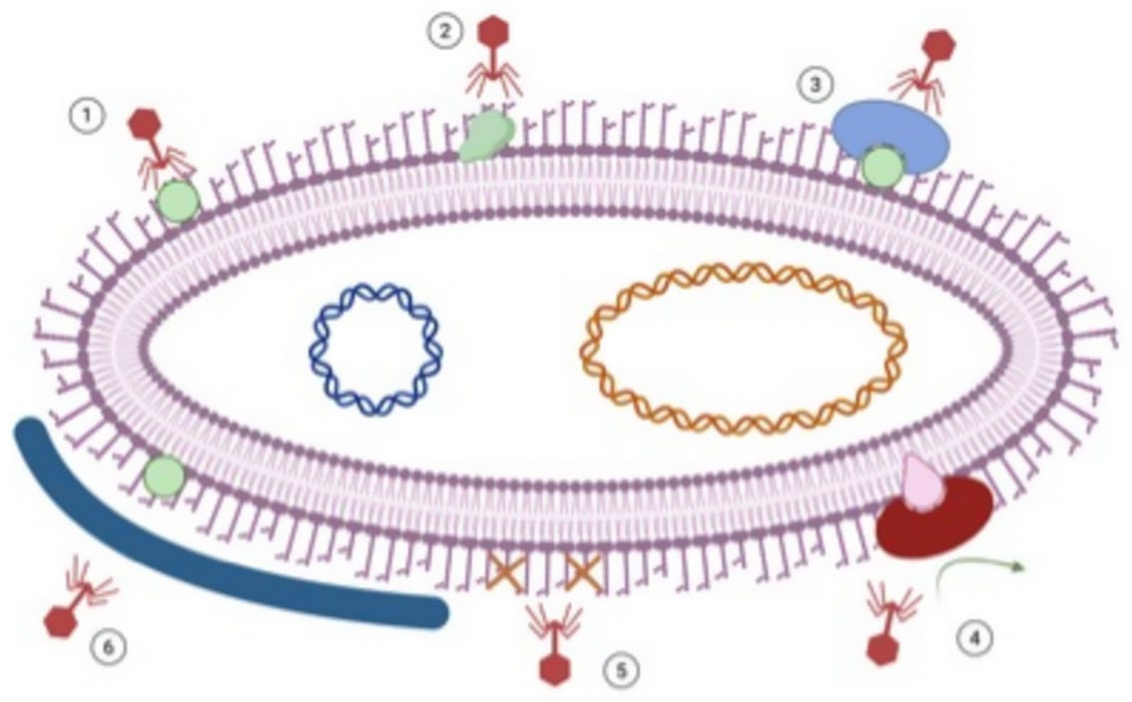
The mechanisms of bacterial resistance to phage attachment. (1) Attachment of phage to its surface receptor on a susceptible host bacterial cell. (2) Modification of the structure of the host receptor. (3) Masking the surface receptor by modifying the expression of the protein that masks it. (4) Competitive inhibition. (5) Loss of lipopolysaccharide and other related receptors. (6) Blocking the receptor via the production of a capsule (Egido et al., 2022).

A particularly important and therapeutically informative resistance mechanism is reversible transposon-mediated regulation of capsule and LOS expression, which enables dynamic switching between phage susceptibility and immune evasion. In this system, non-encapsulated, phage-resistant variants become more vulnerable to complement-mediated killing, whereas encapsulated cells evade host immunity but remain susceptible to phages (Chen et al., 2025). These mechanisms, summarized in Figure 4, illustrate the remarkable evolutionary plasticity of *A. baumannii* and explain why phage monotherapy is inherently vulnerable to resistance when applied in isolation. In response to these resistance barriers, phage-based mitigation strategies have increasingly shifted toward multifaceted and adaptive therapeutic designs. Phage cocktails combining multiple phages that target distinct bacterial receptors represent a central approach to broaden host range, enhance biofilm disruption, and suppress resistance emergence. This strategy has demonstrated strong efficacy against clinically relevant *A. baumannii* lineages; notably, KL2-type strains accounted for 17.7% of isolates nationwide in China and 33.3% of CRAB isolates in Guangdong Province, with an optimized phage cocktail successfully targeting 89.1% of these strains. Importantly, resistance emerging under cocktail treatment was associated with favorable evolutionary trade-offs, including increased antibiotic susceptibility, reduced virulence, and impaired biofilm formation, largely driven by mutations affecting LOS and capsule biosynthesis pathways (Liu et al., 2025).

Phage–antibiotic synergy further enhances therapeutic robustness, as combination regimens consistently demonstrate superior antibacterial activity against XDR strains and reduced resistance selection compared with monotherapies (Rastegar et al., 2024b). Advances in phage engineering provide additional tools to counter resistance, including modification of receptor-binding proteins, expansion of host range, and incorporation of depolymerases to degrade capsules and biofilm matrices. Engineered phages such as Ab105-2phiΔCI404ad, particularly when deployed within rationally designed cocktails, have shown potent antibiofilm and antimicrobial activity (Blasco et al., 2022; Febrianti et al., 2025). Complementary approaches, including phage-curcumin synergy and phage-derived lysins such as P307SQ-8C, further expand the therapeutic arsenal by enabling efficient biofilm eradication and substantial bacterial load reduction *in vitro* and *in vivo* without detectable cytotoxicity (Thandar et al., 2022; Janesomboon et al., 2025). Finally, emerging safety and translational data support the feasibility of resistance-aware phage therapy. Preclinical investigations incorporating transcriptomic profiling of host immune responses indicate controlled immunomodulatory effects, while the existing human evidence base, though still limited, consists primarily of compassionate-use cases and small observational studies, rather than large randomized clinical trials (Li et al., 2024). These reports nonetheless demonstrate the real-world potential of phage-based interventions in resolving otherwise untreatable *A. baumannii* infections. Taken together, effective management of phage resistance in *A. baumannii* will depend on integrative strategies that combine phage cocktails, engineered phages, synergistic adjuvants, and informed consideration of host-pathogen-phage co-evolution, rather than reliance on single-modality phage therapy.

## Phage–antibiotic combination therapy against *Acinetobacter baumannii*

Phage–antibiotic combination (PAC) therapy has emerged as a strategically important approach for combating MDR *A. baumannii*, offering a biologically rational means of enhancing antibacterial efficacy while suppressing resistance evolution (Gordillo Altamirano et al., 2022). Rather than functioning as independent antimicrobial agents, phages and antibiotics frequently engage in cooperative interactions that reshape bacterial susceptibility landscapes, reduce effective drug concentrations, and improve treatment durability. Growing experimental evidence indicates that such synergy is not an isolated phenomenon but a reproducible therapeutic pattern observed across diverse phage–antibiotic pairings and infection models. Early demonstrations of phage–antibiotic synergy (PAS) showed that phage YC#06, when combined with chloramphenicol, imipenem, and cefotaxime, significantly reduced antibiotic requirements while inhibiting biofilm formation *in vitro* (Luo et al., 2022). Importantly, this interaction translated into effective bacterial clearance in a zebrafish infection model, reinforcing the clinical plausibility of PAS beyond controlled laboratory environments (Luo et al., 2022). Similar translational momentum is evident in studies evaluating phage pB23 in combination with meropenem, which eradicated mature biofilms and prevented biofilm establishment in carbapenem-resistant *A. baumannii*. Validation *in* both *ex vivo* pig skin explants and *in vivo* zebrafish infections further highlights the robustness of this strategy across physiologically relevant systems (Luo et al., 2024). Collectively, these findings suggest that PAS may be particularly valuable in biofilm-associated infections, where monotherapies frequently fail due to restricted antibiotic penetration and metabolic heterogeneity within bacterial communities.

Additional phage–antibiotic pairings underscore the functional versatility of this approach. The combination of phage Indie with ceftazidime achieved greater than 85% bacterial reduction while simultaneously suppressing the emergence of phage resistance (Orozco-Ochoa et al., 2025). Notably, phage exposure restored bacterial susceptibility to ceftazidime, illustrating an evolutionarily informed therapeutic advantage whereby resistance to one agent imposes fitness costs that re-sensitize bacteria to another. Comparable outcomes were reported for phage pB3074 combined with cefotaxime and meropenem, which removed mature biofilms and inhibited their formation in both *in vitro* systems and pig skin explant models (Luo et al., 2023). Broad-spectrum synergistic activity has also been documented for phage T1245 paired with multiple antibiotics, including ceftazidime, colistin, imipenem, and meropenem, resulting in substantial reductions in bacterial density and biofilm biomass (Soontarach et al., 2022). Importantly, *in vivo* confirmation of PAS continues to expand; the combination of phage øFG02 with ceftazidime significantly lowered bacterial burden in a murine infection model while promoting the emergence of phage-resistant yet antibiotic-sensitive bacterial populations, a trade-off that may be therapeutically exploitable (Gordillo Altamirano et al., 2022).

Mechanistically, the success of PAS is supported by multiple complementary processes that extend beyond additive killing. Sublethal antibiotic exposure can induce bacterial filamentation, alter cell surface architecture, or increase metabolic activity, thereby enhancing phage adsorption and intracellular replication (Luo et al., 2023). Conversely, phage-mediated selection pressures frequently drive receptor modifications, efflux pump alterations, or capsule loss, adaptations that may restore antibiotic susceptibility or reduce bacterial virulence (Orozco-Ochoa et al., 2025; Gordillo Altamirano et al., 2022). The combined capacity of phages and antibiotics to penetrate and dismantle biofilms further amplifies therapeutic impact, positioning PAS as a particularly attractive strategy for chronic, device-associated, and recalcitrant infections (Luo et al., 2022; Luo et al., 2024; Luo et al., 2023; Soontarach et al., 2022). Despite these promising advances, the efficacy of PAS is highly dependent on treatment design. Optimal outcomes appear contingent upon carefully calibrated phage dosing, as excessively high titers may accelerate resistance selection, whereas insufficient titers risk incomplete bacterial clearance (Luo et al., 2022; Luo et al., 2024). Emerging evidence also suggests that sequential administration, where phage exposure precedes antibiotic therapy, may outperform simultaneous delivery by first destabilizing bacterial defenses and enhancing antibiotic penetration (Erol et al., 2023; Mukhopadhyay et al., 2023). These observations highlight the importance of pharmacodynamic alignment and reinforce the need for standardized therapeutic frameworks. Future investigations should prioritize mechanistic standardization, comparative trial designs, and clinically relevant infection models to accelerate the translation of PAS from experimental validation to routine therapeutic application.

## Host immune interactions and safety considerations

Bacteriophages are increasingly recognized as biologically active agents that interact dynamically with the mammalian immune system rather than functioning solely as passive antibacterial entities. Phages can be detected by host pattern recognition receptors (PRRs), leading to activation of innate immune signaling pathways and cytokine responses that resemble, in part, those elicited by eukaryotic viruses (Górski et al., 2012; Champagne-Jorgensen et al., 2023; Dzananovic et al., 2018). In addition to extracellular recognition, phages may be internalized by immune and non-immune cells, facilitating antigen processing and presentation and promoting the generation of phage-specific antibodies (Champagne-Jorgensen et al., 2023). These interactions position phages as immunologically visible biological agents whose therapeutic activity unfolds within, rather than independently of, host immune networks.

At the immunological level, phage administration has been associated with both pro-inflammatory and regulatory cytokine responses, including induction of IL-1β, IL-6, and IFN-γ alongside IL-10 and IL-4 (Grabowski et al., 2022). However, it is critical to distinguish direct phage-driven immunomodulation from secondary immunological effects resulting from rapid bacterial clearance. In most infection models, reductions in inflammatory markers and tissue damage closely parallel decreases in bacterial burden, indicating that improved inflammatory profiles are predominantly attributable to pathogen elimination rather than intrinsic anti-inflammatory activity of phages. Nonetheless, phages may indirectly shape immune responses by accelerating bacterial clearance kinetics and altering antigen load, thereby influencing the magnitude and duration of host inflammation (Palma and Qi, 2024).

Phages can also interact synergistically with host immunity during infection, particularly by facilitating opsonophagocytic clearance once bacterial surface barriers or biofilms are disrupted. This immune–phage cooperation may enhance therapeutic efficacy but also introduces feedback mechanisms that influence phage persistence and resistance evolution. For example, immune-mediated clearance of phage-sensitive bacterial populations can create selective pressure favoring phage-resistant variants, while immune recognition of phages themselves may limit treatment durability in systemic settings. Thus, immune responses play a dual role, contributing both to bacterial elimination and to the ecological context in which phage–bacteria co-evolution occurs.

A key immunological constraint on phage therapy is the development of phage-specific neutralizing antibodies, which can reduce bioavailability and diminish efficacy over time. The clinical relevance of antibody-mediated neutralization is strongly influenced by route of administration, dosing frequency, and phage type. Neutralizing responses are more pronounced following repeated systemic administration, particularly intravenous delivery, whereas topical, inhaled, or localized applications appear less susceptible due to reduced systemic exposure (Tan et al., 2024; Chechushkov et al., 2021). Lytic phages administered intermittently, or as short courses may also evade rapid immune neutralization compared with prolonged or high-dose regimens. These observations underscore the importance of tailoring dosing strategies, delivery routes, and phage selection to minimize immune-mediated loss of efficacy. Recent advances in formulation engineering provide practical strategies to mitigate immune-mediated limitations of phage therapy. For instance, minimal PEGylation of a lytic bacteriophage targeting multidrug-resistant *A. baumannii* significantly improved pharmacokinetic stability, reduced immunogenicity, and enhanced therapeutic efficacy in vivo, demonstrating that rational chemical modification can enhance phage bioavailability while preserving antibacterial activity (Choi et al., 2026).

From a safety perspective, phage therapy has demonstrated a consistently favorable profile in both preclinical models and human studies. Across multiple investigations, phages were well tolerated following oral, intravenous, and topical administration, with minimal adverse effects reported (Chung et al., 2023; Gangwar et al., 2022; Ibrahim et al., 2025; Nie et al., 2025). Long-term or high-dose exposure in animal models did not result in significant toxicity, pathological immune activation, or systemic organ damage, supporting the overall biocompatibility of phage-based interventions (Gangwar et al., 2022). Nevertheless, specific safety considerations remain, particularly the potential release of endotoxins following rapid bacterial lysis and the emergence of phage-resistant bacterial subpopulations, both of which necessitate careful therapeutic design, controlled dosing, and clinical monitoring (Colavecchio et al., 2017).

Repeated or extended phage therapy may also provoke measurable immune changes, such as splenic enlargement or transient cytokine elevation, reflecting immune engagement rather than overt pathology (Tan et al., 2024). While these responses may limit efficacy in some contexts, they also highlight the need for optimized regimens, including phage rotation, cocktail strategies, engineered phages, or combination therapies, to balance antibacterial efficacy with immune tolerance.

Accumulated experimental and clinical evidence supports phage therapy as a generally safe and promising alternative or adjunct to antibiotics, particularly for multidrug-resistant infections. However, a key unresolved challenge is the rational integration of immune dynamics into phage therapy design, including prediction of immune neutralization, management of inflammatory consequences of rapid bacterial lysis, and understanding how host immunity shapes phage resistance trajectories. Addressing these immunological dimensions will be essential for transitioning phage therapy from compassionate-use success to durable, standardized clinical practice (Nie et al., 2025; Chung et al., 2023; Ibrahim et al., 2025; Alaaeddine et al., 2025).

## Regulatory, manufacturing, and translational challenges

Good Manufacturing Practice (GMP) is fundamental to ensuring the quality, safety, and efficacy of bacteriophage-based medicinal products. Although production frameworks are often compared to those used in vaccine manufacturing, phage therapeutics differ fundamentally in their replicative capacity, narrow host specificity, and evolutionary adaptability, features that complicate their regulation within conventional biologics paradigms (Bretaudeau et al., 2020; Suleman M. et al., 2025; Suleman A. R. et al., 2025). Accordingly, GMP compliance necessitates contamination-free production environments, reproducible manufacturing processes, and rigorous quality control aligned with internationally recognized regulatory standards (Pirnay et al., 2024; Merabishvili et al., 2009). From a regulatory standpoint, critical release and comparability assays include sterility and pyrogenicity testing to exclude microbial contaminants and endotoxins, stability assessments under defined storage conditions, and genomic confirmation of a strictly lytic lifestyle characterized by the absence of toxin-encoding, antimicrobial resistance, and lysogeny-associated genes. Morphological validation through electron microscopy, together with standardized potency and host-range evaluations, including the assessment of phage-antibiotic synergy, also remains central to regulatory approval (Merabishvili et al., 2009; Sisakhtpour et al., 2022).

Although deeper proteomic profiling, adsorption kinetics, and exploratory host-range mapping provide valuable insights during early research phases, prioritizing regulator-critical assays is essential for enabling scalable GMP production. Nevertheless, achieving strict standardization remains inherently challenging, as batch-to-batch consistency is influenced by biological variability, reliance on bacterial production hosts, fluctuations in phage yield, and the adaptive nature of phages relative to small-molecule antibiotics (Mohammadi et al., 2025). These manufacturing constraints directly contribute to the persistent scarcity of large randomized controlled trials (RCTs), as multicenter trials require highly reproducible products with minimal batch variability, yet biologically driven fluctuations in phage amplification, purification efficiency, and host-strain performance complicate cross-site comparability and regulatory acceptance (Malik et al., 2017). In the absence of universally harmonized manufacturing standards, protocol alignment across institutions becomes difficult, increasing trial costs and logistical complexity while discouraging large-scale industrial sponsorship.

Regulatory complexity further constrains clinical translation. In both the European Union and the United States, bacteriophages are classified as biological medicinal products, requiring extensive preclinical validation and phased clinical trials under drug-centric regulatory frameworks (Pelfrene et al., 2021; Verbeken et al., 2014). While appropriate for fixed pharmaceutical agents, these frameworks remain poorly aligned with the intrinsic flexibility required for phage therapy, particularly when rapid phage substitution or customization is necessary to address evolving bacterial targets. Even minor modifications in phage composition may trigger additional regulatory review, creating administrative and financial burdens that delay trial initiation and deter investment in adequately powered RCTs. This tension between therapeutic adaptability and regulatory expectations is further reflected in manufacturing strategies that follow either personalized approaches, tailored to individual patients and bacterial isolates, or standardized, off-the-shelf formulations intended for broader clinical use (Pirnay et al., 2024).

Personalized phage therapy offers precision and responsiveness but conflicts with regulatory requirements for product uniformity, reproducibility, and blinded allocation, whereas standardized formulations improve scalability yet may demonstrate reduced efficacy against genetically diverse clinical isolates, complicating endpoint interpretation (Yang et al., 2023). Additional translational challenges arise from the emergence of bacteriophage-insensitive mutants (BIMs), which complicate treatment durability and trial design. Resistance development during therapy underscores the limitations of static phage formulations and reinforces the need for adaptive therapeutic strategies; however, such adaptability inherently challenges traditional RCT frameworks built around fixed interventions and predefined endpoints. Although phage cocktails and genetically engineered phages can mitigate BIM emergence by broadening host range and enhancing antibacterial robustness, these approaches introduce further regulatory complexity, as modifications in phage composition or genetic structure may necessitate repeated regulatory reassessment (Kolenda et al., 2022; Ibrahim et al., 2025). Combination therapy presents an additional layer of regulatory difficulty. While phage–antibiotic synergy enhances antibacterial efficacy and reduces resistance selection, multi-component regimens raise challenges related to dosing justification, pharmacodynamic interactions, attribution of therapeutic benefit, and product approval (Roque-Borda et al., 2025). Collectively, regulatory rigidity, manufacturing variability, lack of standardized production and clinical protocols, and persistent barriers to cross-site trial harmonization explain why the current evidence base remains dominated by compassionate-use reports and small cohort studies rather than adequately powered randomized controlled trials.

Despite these limitations, accumulating preclinical and clinical data support the therapeutic promise of phage-based interventions against multidrug-resistant *A. baumannii*, with studies consistently reporting favorable safety profiles, reductions in bacterial burden, improved clinical recovery, and enhanced efficacy when phages are combined with antibiotics (Shivaswamy et al., 2016; Kusradze et al., 2016). Nevertheless, the limited number of large RCTs continues to restrict definitive conclusions regarding optimal dosing strategies, regulatory approval pathways, and standardized clinical implementation. In parallel, emerging non-antibiotic phage-derived modalities, including depolymerases, endolysins, engineered phages, photosensitizer-conjugated phages, and rationally designed phage cocktails, have demonstrated strong preclinical efficacy against biofilm-associated *A. baumannii* infections and represent promising strategies for enhancing therapeutic robustness while limiting resistance development (Mendes et al., 2023; Wang et al., 2025a, b). These approaches differ substantially in regulatory classification and translational pathways: depolymerases and endolysins are typically regulated as protein therapeutics, whereas engineered phages introduce additional considerations related to genetic modification, environmental release, and long-term safety monitoring (Taati Moghadam et al., 2025). Although GMP compliance provides a critical foundation for safe phage deployment, overcoming regulatory fragmentation, manufacturing constraints, including yield variability and host strain standardization, resistance dynamics, and persistent gaps in high-level clinical evidence will be essential for routine clinical integration. Future progress will likely depend on globally harmonized GMP frameworks, adaptive regulatory pathways, and innovative platform trial designs capable of balancing therapeutic flexibility with methodological rigor.

## Conclusion

*A. baumannii* remains a critical threat in healthcare settings due to its exceptional capacity for environmental persistence, biofilm formation, and rapid acquisition of multidrug resistance. As conventional antibiotics continue to lose effectiveness, bacteriophage-based strategies have re-emerged as promising complementary approaches grounded in well-defined microbiological and evolutionary principles. Current evidence demonstrates that lytic phages, phage cocktails, phage-derived depolymerases and endolysins, and engineered phage systems can reduce bacterial burden, disrupt biofilms, and impose fitness trade-offs on resistant subpopulations, often enhancing antibiotic activity. However, therapeutic outcomes are highly context-dependent and influenced by formulation strategy, delivery route, dosing, and host immune responses. Importantly, many reported benefits reflect secondary consequences of bacterial clearance rather than direct immunomodulatory effects, underscoring the need for careful interpretation of experimental and clinical data. Despite growing preclinical support and increasing compassionate-use experience, significant challenges remain, including host-range limitations, resistance evolution, formulation stability, immune neutralization, and regulatory and manufacturing constraints. Addressing these barriers will require mechanism-driven phage selection, standardized characterization frameworks, and integrative translational strategies that align microbiological insight with clinical and regulatory requirements. Overall, bacteriophage-based interventions represent a valuable adjunct to existing antimicrobial strategies rather than a standalone solution. Continued interdisciplinary research and well-designed clinical studies will be essential to define their optimal role in the treatment of antibiotic-resistant *A. baumannii* infections.
